# MPC-n (IgG) improves long-term cognitive impairment in the mouse model of repetitive mild traumatic brain injury

**DOI:** 10.1186/s12916-023-02895-7

**Published:** 2023-05-30

**Authors:** Chaonan Zhang, Cheng Wei, Xingqi Huang, Changxin Hou, Chuan Liu, Shu Zhang, Zilong Zhao, Yafan Liu, Ruiguang Zhang, Lei Zhou, Ying Li, Xubo Yuan, Jianning Zhang

**Affiliations:** 1 Key Laboratory of Post-Neurotrauma Neuro-Repair and Regeneration in Central Nervous System, Ministry of Education and Tianjin City, Department of Neurosurgery, Tianjin Neurological Institute, Tianjin Medical University General Hospital, Tianjin, 300052 China; 2 Tianjin Key Laboratory of Composite and Functional Materials, School of Materials Science and Engineering, Tianjin University, Tianjin, 300072 China

**Keywords:** Repetitive mild traumatic brain injury, Immunoglobulin, Cognitive impairment, Neuro-inflammation

## Abstract

**Background:**

Contact sports athletes and military personnel who suffered a repetitive mild traumatic brain injury (rmTBI) are at high risk of neurodegenerative diseases such as advanced dementia and chronic traumatic encephalopathy (CTE). However, due to the lack of specific biological indicators in clinical practice, the diagnosis and treatment of rmTBI are quite limited.

**Methods:**

We used 2-methacryloyloxyethyl phosphorylcholine (MPC)-nanocapsules to deliver immunoglobulins (IgG), which can increase the delivery efficiency and specific target of IgG while reducing the effective therapeutic dose of the drug.

**Results:**

Our results demonstrated that MPC-capsuled immunoglobulins (MPC-n (IgG)) significantly alleviated cognitive impairment, hippocampal atrophy, p-Tau deposition, and myelin injury in rmTBI mice compared with free IgG. Furthermore, MPC-n (IgG) can also effectively inhibit the activation of microglia and the release of inflammatory factors.

**Conclusions:**

In the present study, we put forward an efficient strategy for the treatment of rmTBI-related cognitive impairment and provide evidence for the administration of low-dose IgG.

**Supplementary Information:**

The online version contains supplementary material available at 10.1186/s12916-023-02895-7.

## Background

Traumatic brain injury (TBI) is a common and serious neurological disease, which contributes to approximately $400 billion annually [1]. The number of new TBI patients is as high as 50 million to 60 million each year, and 80–90% of them are mild TBI (mTBI) [2, 3]. In particular, repetitive mild traumatic brain injury (rmTBI) can contribute to chronic traumatic encephalopathy (CTE) due to the continuous accumulation of damage. CTE is mainly characterized by phosphorylated-Tau (p-Tau) deposition, microglial activation, and white matter rarefaction [4–6].

The highest incidence of rmTBI patients are among contact sports athletes, military veterans, and elderly people who have fallen over the years [4, 7]. Because of the mild symptoms and the late onset, the consultation rate of rmTBI is low and the treatment is always delayed.

Immunoglobulin (IgG) is a polyclonal product purified from human serum and has been used as an effective first-line drug for various neurological diseases, such as Guillain Barre syndrome, chronic inflammatory demyelinating polyneuropathy, and multifocal motor neuropathy [8]. Since IgG can directly target the immune system and neurons, IgG has also shown a potential in the treatment of ischemic stroke [9, 10]. In addition, in preclinical and clinical trials of mild to moderate Alzheimer’s disease (AD), IgG was reported to efficiently reduce amyloidosis (Aβ) and modulated the neuroimmune response, as well as remit brain atrophy in patients [11, 12]. In a study of closed cranial trauma, high doses of IgG (600 mg/kg) were shown benefits in improving behavioral and cognitive function in mice with a single impact [13].

However, high dose use of IgG was limited considering safety and feasibility. Many clinical trials have found that patients treated with high-dosage IgG tend to suffer side effects, such as thrombosis and anaphylaxis [8, 14]. In recent years, nanocapsules were used to improve the delivery efficiency and reduce the effective therapeutic dose of drugs due to their excellent biocompatibility and blood–brain barrier (BBB) permeability [15]. We have previously demonstrated that 2-methacryloyloxyethyl phosphorylcholine (MPC) synthesized with the MPC monomer and ethylene glycol dimethyl acrylate (EGDMA) crosslinker, as a choline and acetylcholine analog, can be taken up and transferred by high-affinity choline transporter (ChTs) receptors of endothelial cells from the blood into the brain parenchyma [16].

Although IgG has been extensively studied in a variety of neurological disorders, its efficacy in cognitive impairment in rmTBI has been rarely studied. In the present study, MPC-capsuled IgG (MPC-n (IgG)) was used to treat long-term cognitive impairment in rmTBI mice. We first demonstrated the specific accumulation of MPC-n (IgG) in the cortex and hippocampus of rmTBI mice, which confirmed that MPC-n (IgG) increased the BBB penetration and drug delivery efficiency. Next, we determined p-Tau deposition, hippocampal atrophy, cognitive function, and microglial activation in rmTBI mice during the chronic phase. Our study indicates that MPC-n (IgG) can effectively ameliorate cognitive dysfunction and neuroinflammation in rmTBI mice, which provides a potential strategy for the application with a low dose of IgG.

## Methods

### Materials

IgG was purchased from Solarbio. N-(3-Aminopropyl) methacrylamide hydro-chloride (APM) was purchased from Macklin. 2-Methacryloyloxyethyl phosphorylcholine (MPC), ethylene glycol dimethyl acrylate (EGDMA), fluorescein isothiocyanate (FITC), and 2-(1E, 3E, 5E)-5-(3-(6-(2, 5-dioxopyrrolidin-1-yl) oxy)-6-oxohexyl)-1 (Cy5.5) were purchased from Sigma-Aldrich. Ammonium persulfate (APS), N, N, N′, N′-tetramethylethylenediamine (TEMED) were obtained from Alfa Aesar. All reagents were used without purification.

### Synthesis of MPC-n (IgG)

The preparation principle of nano-microcapsules is to carry out in situ free radical polymerization on the surface of IgG. The specific process is as follows: dissolving IgG in 1 mL phosphate buffer (PBS, pH 7.4), adding APM, and MPC to mix evenly, and then adding cross-linking agent EGDMA, molar ratios of free IgG to APM to MPC to EGDMA = 1: 300: 7000: 600. After mixing for 10 min, the catalyst TEMED and initiator APS (APS: IgG = 300, n/n; TEMED: APS = 2:1, w/w) [16, 17]. The reaction will last 4 h. At the end of the reaction, the nanocapsules with MPC as monomers were obtained. After that, we use dialysis bags (MWCO: 8000–14,000, Solarbio) to remove the free monomer. It was used for dialysis in phosphate buffer saline (10 mM PBS, pH = 7.4). The fresh PBS solution was replaced every 6 h, and the nanocapsules solution was obtained after 48 h of dialysis. And then, to remove unencapsulated proteins, we passed the solution through a hydrophobic interaction column (Phenyl-Sepharose CL-4B, Solarbio, laboratory reagent). The purified nanocapsules were stored at 4 °C.

### Characterization of MPC-n (IgG)

The particle size of the nanoparticle solution after 1 mL (1 mg/mL) dialysis was measured by Brookhaven's BI-90 Plus Zeta PALS analyzer [18]. The morphology of nanocapsules was characterized by using the JEM-2100F field transmission electron microscope (TEM) with an acceleration voltage of 200 kV. The solution of nanocapsules (0.01 mg/mL) was dripped onto the copper mesh and it was stained with 2% (w/v) phosphotungstic acid solution, washed with deionized water, fully dried, and then observed under TEM. The chemical groups on the surface of nano-microcapsules were analyzed by the Fourier transform infrared (FT-IR). The freeze-dried sample was mixed with potassium bromide and fully ground, and IgG, MPC-n (IgG) and gel without IgG were prepared for scanning [19]. The scanning range is 400–4000 cm^−1^. The freeze-dried sample MPC, IgG, and MPC-n (IgG) were dissolved in 0.6 mL D_2_O, and proton nuclear magnetic resonance (^1^H NMR) spectra were measured using AVANCE IIITM HD 400 MHz NanoBAY (Bruker). We can also scan them with a UV–vis spectrophotometer (AOE instruments, A360 spectrophotometer). The range is 200^−^500 nm. The freeze-dried MPC-n (IgG) was added into the solution of PBS (pH = 7.4) to form 1 mg/mL, and the same in H_2_O, DMEM, and serum at 37 °C for 48 h. The stability of MPC-n (IgG) was quantified using the scattering light intensity ratio I/I0 by dynamic light scattering (DLS) [20]. Because the crosslinker has an ester group with pH response, we set up two groups of nanocapsules under different pH (pH = 6.5, pH = 7.4) and measured the light scattering intensity *I*_0_ of the two groups of solutions respectively. After the determination, both groups of samples were incubated at 37 °C for 60 min. In the process of incubation, the DLS intensity *I* was measured by the solution of nanocapsules at different time points, and the enzymatic degradation kinetics of nanocapsules were detected by *I*/*I*_0_.

### Animals

Adult male C57BL/6 J mice (aged 8–10 weeks old, weighing 20–25 g) purchased from Hfk Bioscience company (Beijing, China) were housed at the Experimental Animal Laboratories of Tianjin Neurological Institute. They were randomly fed food and water with a 12-h light/dark cycle. All experimental operations were allowed by the Animal Ethics Committee of Tianjin Medical University.

### Experimental rmTBI model

The mice were anesthetized with 4.6% isoflurane and fixed to an acrylic mold. A concave metal disc was placed caudal to bregma on the shaved head of mice, and the impounder tip of controlled cortical impact (electronic CCI model 6.3, American Instruments, Richmond, VA, USA) was positioned at the center of the disc surface, which is discharged at 5 m/s with a head displacement of 5 mm. Mice were divided into four groups: sham, rmTBI, rmTBI + IgG, and rmTBI + MPC-n (IgG) group. Injured mice were impacted 4 times with a 48-h interval. The sham group underwent the same operating procedures without any impact. After the last impact, the rmTBI mice were respectively administrated IgG (600 mg/kg) and MPC-n (IgG) (120 mg/kg) through the tail veil.

### In vivo* distribution, imaging, and quantification*

The distribution of Cy5.5-labeled MPC-n (IgG) and IgG in mice was observed using the in vivo imaging system (IVIS Lumina II, PerkinElmer, USA) 2, 6, and 24 h post-injection. Mouse brain tissues of all groups were collected 24 h post-injection. Fluorescence intensity values were acquired and analyzed using the Living Image software version 3.1 (Caliper Life Sciences) [17].

### Immunofluorescence staining

Mouse brains were harvested following cardiac perfusion and fixed in 4% PFA for 24 h. The dehydrated tissues were frozen rapidly in liquid nitrogen and sliced into 6-μm-thick sections with the freezing microtome (Leica CM 1950). Sections were blocked with 0.3% TritonX and 5% Albumin Bovine V for 1 h. Sections were incubated with primary antibodies overnight at 4 °C (Iba1, 1:500, Abcam; Phospho-Tau, 1:200, Cell Signaling Technology; Phospho-p38 MAPK, 1:200, Cell Signaling Technology). After being washed in PBS, sections were incubated with Alexa Fluor conjugated secondary antibodies (AlexaFluor 488/555, 1:500, Life Technologies) and stained with 4′,6-diamidino-2-phenylindole (DAPI, Sigma).

### Western Blot

Brain tissues were obtained at 28 DPI and total protein was lysed with RIPA lysis buffer (Solarbio), PMSF (Solarbio), and phosphatase inhibitor (Sigma). Equally loaded proteins were electrophoretically separated on 10% and 15% SDS-PAGE gels and then transferred to PVDF membranes (Millipore). After being blocked by silk milk, membranes were incubated with primary antibodies for Phospho-Tau, Iba1, Phospho-p38 MAPK (1:1000, Cell Signaling Technology), and GAPDH (1:1000, Abcam). Horseradish peroxidase-conjugated secondary antibodies were used as a chromogenic reagent (1:5000, Zhongshanxinqiao).

### Transmission electron microscope (TEM)

The cortex and hippocampus of mice were fixed in 2.5% glutaraldehyde for 24 h and in 1% osmium tetroxide for 1.5 h at 4 °C. After dehydrated, the tissues were cut into ultra-thin slices and observed under transmission electron microscopy as previously described (TEM, HIT CHI-HT7700) [21].

### Magnetic resonance imaging (MRI)

Mice were anesthetized with isoflurane in a 9.4 T small-bore animal scanner (Bruker bio spec 94/30 USR) at 28 DPI and 42 DPI. The body temperature of mice was monitored and maintained at 37 ± 1° C. T2-weighted images were captured according to the following parameters: repetition time = 2500 ms, echo time = 33 ms, rare factor = 8, the field of vision = 20 × 20 mm, matrix size = 256 × 256, slice thickness = 0.5 mm, scanning time: 2 min 40 s [22].

### Morris water maze test

Morris water maze test was used to evaluate the learning ability and spatial memory of mice. The mice at 28 DPI were placed into the pool from four quadrants to search for the underwater platform in the 90 s. After 90 s, mice that had not found the platform were guided to the platform and stayed for 20 s. Four trials per day for six consecutive days later, the platform was removed on the testing day, and the computer recorded the swimming track, dwelling time, and path length of mice [23].

### Cytokine quantification by Array

Mouse brains of all groups were used for cytokine profiling. The relative levels of different cytokines were assessed by Proteome Profiler Mouse Cytokine Array Panel A (R&D Systems). The densitometry of each spot was measured using the ChemiDo XRS + imaging system (Bio-Rad, CA, USA), and the pixel density was evaluated by ImageJ Software.

### Sampling and preparation of mouse brain tissue samples

RNA samples were sent to Shanghai Bohao Biotechnology Co., Ltd., China for cRNA library preparation and RNA sequencing. Total RNA from the samples was extracted by the Animal Total RNA Extraction Kit (Magnetic Bead method, MJYHIVD). Among them, RNA quantity and quality were assessed using Agilent 2100 Bioanalyzer (Agilent Technologies, Santa Clara, CA, US) and RNAClean XP Kit (Cat A63987, Beckman Coulter, Inc. Kraemer Boulevard Brea, CA, USA) and RNase-Free DNase Set (Cat#79,254, QIAGEN, GmBH, Germany) were used for purification. All samples used Illumina NovaSeq6000 sequencer, model: PE150.

### Gene expression and differential gene acquisition

FASTQ data were processed using Seqtk (https://github.com/lh3/seqtk) to extract qualified raw read sub-data (the amount of data is about 6G/sample, and the ratio of base quality greater than 20 (Q20) in each direction is no less than 90%). We used the spliced mapping algorithm of Hisat2 (version: 2.0.4) [24] to perform genome mapping on the preprocessed reads, where the mapped genome is “GRCm38.p4 (mm10)”(ftp://ftp.ensembl.org/pub/release83/fasta/mus_musculus/dna/Mus_musculus.GRCm38.dna.primary_assembly.fa.gz), the parameter defaults. Then “Stringtie” (version: 1.3.0) was used to count the “Fragments” of the mapped genes [25, 26], and after normalization using the TMM method [27], the FPKM value of each gene was calculated using the perl script.

All downstream analyses were performed in R version 3.6.3. “edgeR” was used to screen for differential genes between samples. Genes with log2|FC|> 0.2 and *p* < 0.05 identified were identified as differentially expressed genes (DEGs) [28]. Using “umsp” (version 0.2.7.0) to observe heterogeneity between data samples, DEG volcano plots and heatmaps were visualized using the “ggplot2” and “ComplexHeatmap” packages, respectively.

### Functional enrichment analysis

Gene Ontology (GO) [29] and KEGG enrichment analysis [30] was conducted to detect those DEGs’ function. The “GOplot” package and “cluster profiler” are used to visualize the enrichment results.

### Statistical analysis

All the data were analyzed by Prism 9 (GraphPad Software, San Diego, USA. One-way analysis of variance (ANOVA) followed by Tukey’s post hoc test. All data were presented as mean ± standard error of the mean, and *P*-values less than 0.05 were deemed statistically significant.

## Results

### Characterization of MPC-n (IgG)

**Fig. 1:**
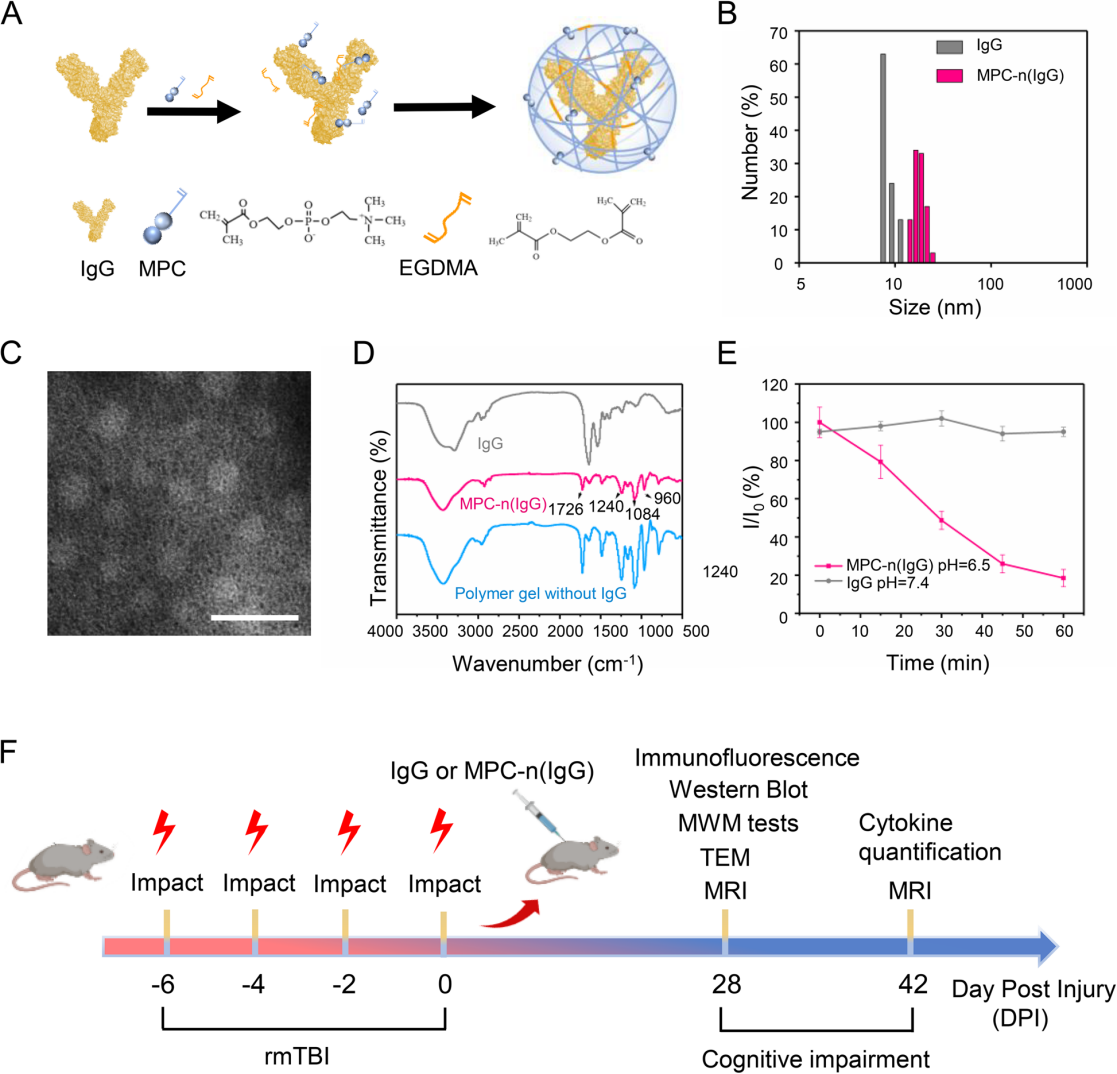
Characterization of MPC-n (IgG). **A** Schematic for the synthesis of MPC-n (IgG). **B** Size of IgG and MPC-n (IgG) measured by dynamic light scattering. **C** Representative transmission electron micrographs of MPC-n (IgG). Scale bar = 50 nm. **D** FT-IR spectra of free IgG, polymer gels without IgG, and MPC-n (IgG) after lyophilization. **E** Release of MPC-n (IgG) of different pH (6.5 and 7.4) measured by dynamic light scattering. **F** Flow chart showing IgG and MPC-n (IgG) administration and experimental design

Nanocapsules are IgG encapsulated by MPC monomer, and then MPC-n (IgG) is formed by adding pH-responsive cross-linking agent EGDMA under the principle of in situ radical polymerization (Fig. 1A). The particle size of the nanocapsules is about 18 nm (± 2 nm). Compared with the particle size of IgG, the particle size of nanocapsules is about 18.5 nm, which increases obviously. The reason for the increase in particle size is that a polymer shell coated with protein is formed on the surface of IgG by in situ free radical reaction, which makes the whole spherical particles larger, and the experimental results are consistent with the analysis, which proves the successful preparation of nanocapsules. This is consistent with the results shown in the TEM photos that the nanocapsules are more regularly spherical (Fig. 1B, C). The surface morphology of the dialyzed nano-microcapsules was analyzed by TEM. From the TEM photos of nanocapsules, we can see that the morphology of MPC-n (IgG) is mostly spherical particles. Compared with the scale, we can see that the particle size is about 20 nm, which is consistent with the particle size measured by our particle size meter. The formation of spherical particles is mainly due to the negative charge of protein in phosphate buffer, while the positively charged monomer APM is a kind of acrylic hydrochloride. Through the interaction of static electricity and hydrogen bond, APM, MPC monomer, and cross-linking agent carry out in situ free radical polymerization on the protein surface under the action of the initiator to form a three-dimensional network structure. The research group proved earlier that if the monomer does not form a shell structure on the protein surface, only the polymerization between monomers will not form a spherical structure, which further illustrates the formation of spherical nanocapsules.

From the FT-IR (Fig. 1D), the absorption peaks identified by 1726 cm^−1^, 1240 cm^−1^, 1084 cm^−1^, and 960 cm^−1^ belong to the ester absorption peak of-COOCH_2_-, the absorption peak of *P* = O, P-O-C, and the antisymmetric stretching vibration peak of C-N. Because the polymer material on the surface of MPC-n (IgG) is mainly PMPC [31], the appearance of these four peaks verifies its existence, while the composition of other monomers and cross-linking agents is difficult to see, mainly due to the small proportion added.

In the ^1^H NMR spectra (Additional file 1: Fig. S1A), the main proton peaks of MPC-n (IgG) were located at 1.8, 3.1, and 4.1–4.3 ppm, which corresponded to the characteristic peaks of poly 2-methacryloyloxy ethyl phosphorylcholine (PMPC). Because the protein has a characteristic peak in 280 nm, in the ultraviolet spectrum (Additional file 1: Fig. S1B), IgG and MPC-n (IgG) will have a characteristic peak in 280 nm, while gel without IgG will not have a characteristic peak, which proves that nanocapsules do contain IgG.

We further confirmed the degradation behavior of MPC-n (IgG) by DLS (Fig. 1E). Compared with pH = 7.4, in the environment of pH = 6.5, with the extension of culture time, *I*/*I*_0_ of MPC-n (IgG) gradually decreased, indicating that the particle size of MPC-n (IgG) was decreasing, which further confirmed that it could be degraded.

To investigate the stability of MPC-n (IgG), it was dissolved in H_2_O, PBS, DMEM, and serum at 37 °C [17], and the size of MPC-n (IgG) was measured by DLS for 48 h. As shown in Additional file 1: Fig. S1C, the MPC-n (IgG) remained stable in H_2_O, PBS, DMEM, and serum at 37 °C for 48 h. These findings suggested that the encapsulation of IgG enabled controlled release and enhanced its stability. The modeling process and disease progression of rmTBI are shown in Fig. 1F.

### MPC-n (IgG) promotes IgG accumulation in rmTBI mice

**Fig. 2:**
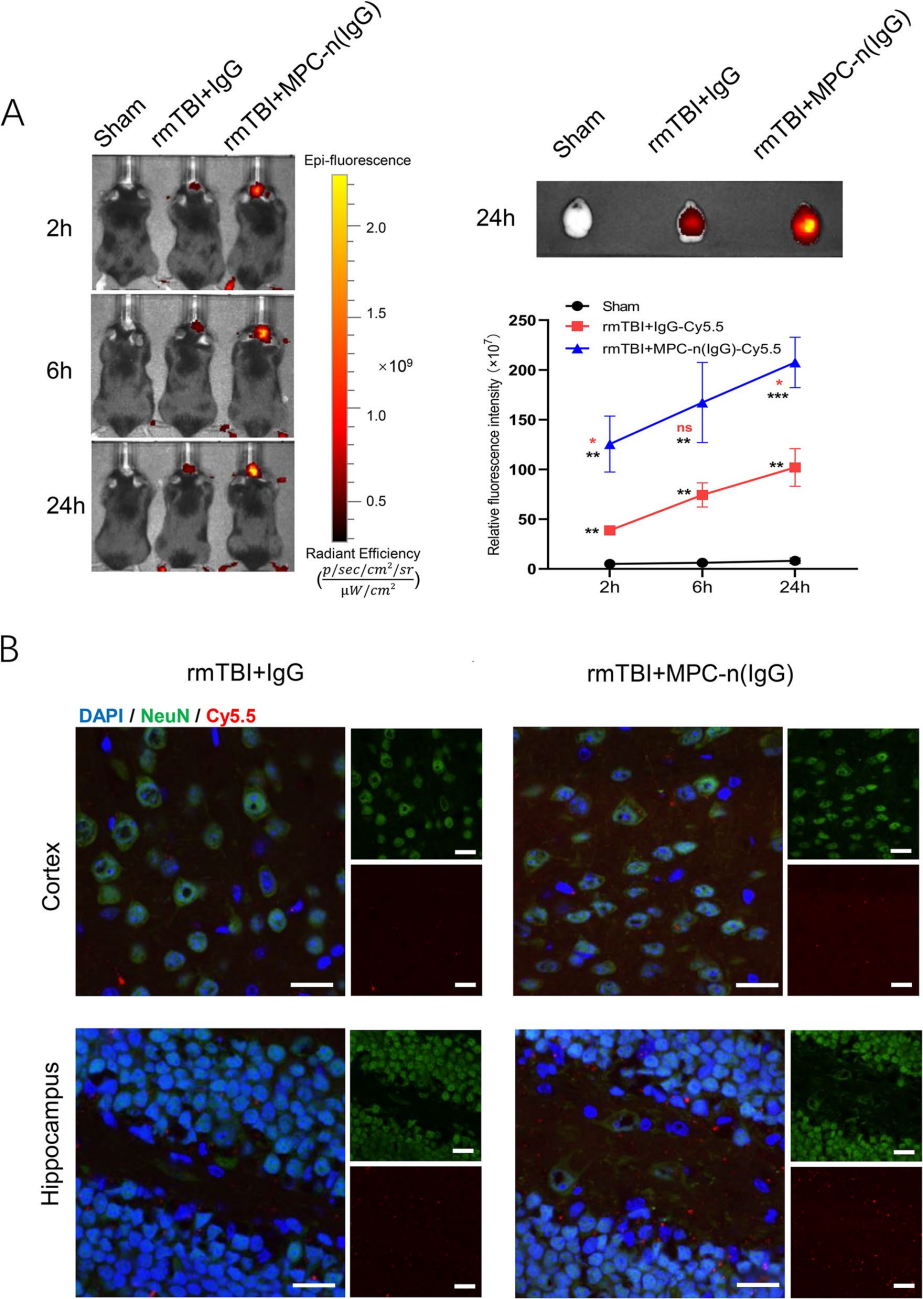
The selective accumulation of MPC-n (IgG) in rmTBI mice. **A** In vivo imaging exhibited the fluorescence distribution of cy5.5 signal in the sham, rmTBI + IgG, rmTBI + MPC-n (IgG) group at 2, 6, and 24 h after the injection in rmTBI mice (*n* = 3). **B** The confocal microscope displayed the spatial distribution of Cy5.5 in the cortex and hippocampus 24 h post injection. Scale bar = 20 μm

Given the good stability and BBB targeting of microcapsules, we first assessed the delivery capacity of MPC-n (IgG) in rmTBI mice. IgG and MPC-n (IgG) coupled with Cy5.5 fluorescence were intravenously injected into rmTBI mice after the last impact. In a vivo imaging system, it exhibited a higher Cy5.5 signal in the mice brains of the MPC-n (IgG) group compared to the IgG group at 2 h, 6 h, and 24 h post-injection (Fig. 2A). In addition, we obtained the mouse brain tissue at 24 h for fluorescence detection, which displayed a consistent result. To further investigate the spatial differences in IgG distribution, the distribution of Cy5.5-coupled IgG and MPC-n (IgG) 24 h post-injection was observed under a confocal microscope, which showed that MPC-n (IgG) was more significantly distributed in the cortex and hippocampus compared to the free IgG (Fig. 2B). These results illustrated that MPC-n (IgG) significantly enhanced BBB permeability of IgG and increased the specific accumulation in the cortex and hippocampus of rmTBI mice.

### MPC-n (IgG) improves cognitive impairment and hippocampal atrophy

**Fig. 3:**
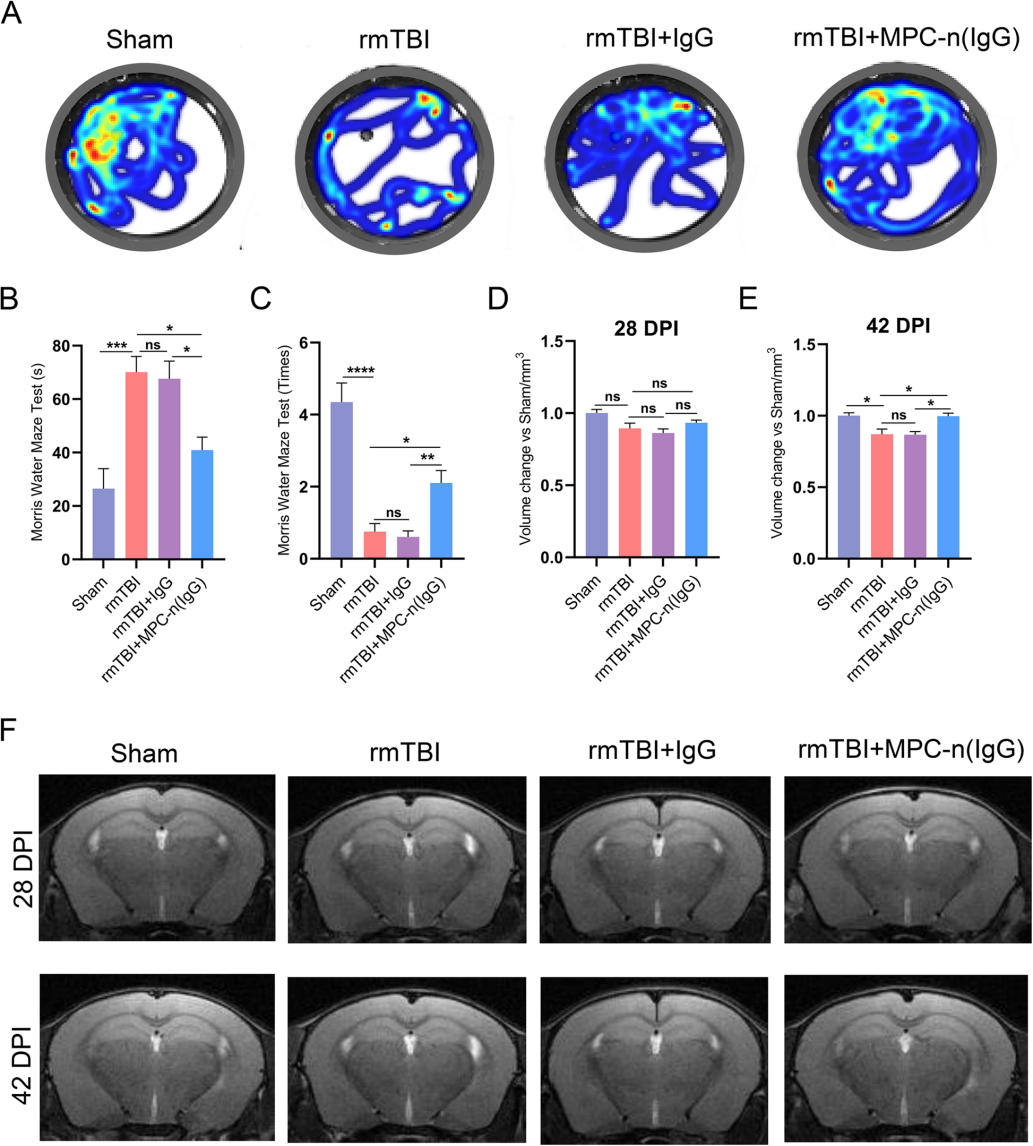
MPC-n (IgG) improves cognitive impairment and hippocampal atrophy. **A** Heat map of the trajectory in the Morris water maze test (*n* = 8). **B** The first latency duration to pass over the platform and **C** the numbers of crossing the platform. **D** The statistics of hippocampal volume change fold at 28 DPI and **E** 42 DPI (*n* = 5–6). **F** The representative magnetic resonance images at 28 and 42 DPI

The main pathological manifestation of rmTBI is long-term cognitive dysfunction. Thus, Morris water maze was performed to determine spatial cognition and memory function in rmTBI mice at 28 DPI [32]. On the testing day, compared with the IgG group, the MPC-n (IgG) group had longer dwelling time in the target quadrant (Fig. 3A), shorter latency to first target-site (Fig. 3B), and increasing number of platform-site crossovers (Fig. 3C). These results suggested that MPC-n (IgG) can significantly improve long-term cognitive and memory function more than free IgG.

Due to the sensibility of MRI in anatomical detail, it was performed to further measure the hippocampal volume changes. Although there was no significant statistical difference among the groups at 28 DPI (Fig. 3D), MPC-n (IgG) showed a therapeutic effect in hippocampal atrophy at 42 DPI (Fig. 3E), while IgG had no distinct benefit at each time point. These results indicated that the hippocampal lesions were still progressing after rmTBI in the long term, and MPC-n (IgG) could ameliorate cognitive and memory dysfunction through mitigating hippocampal atrophy.

### Administration of MPC-n(IgG) alleviates Tau deposition and myelin injury

**Fig. 4:**
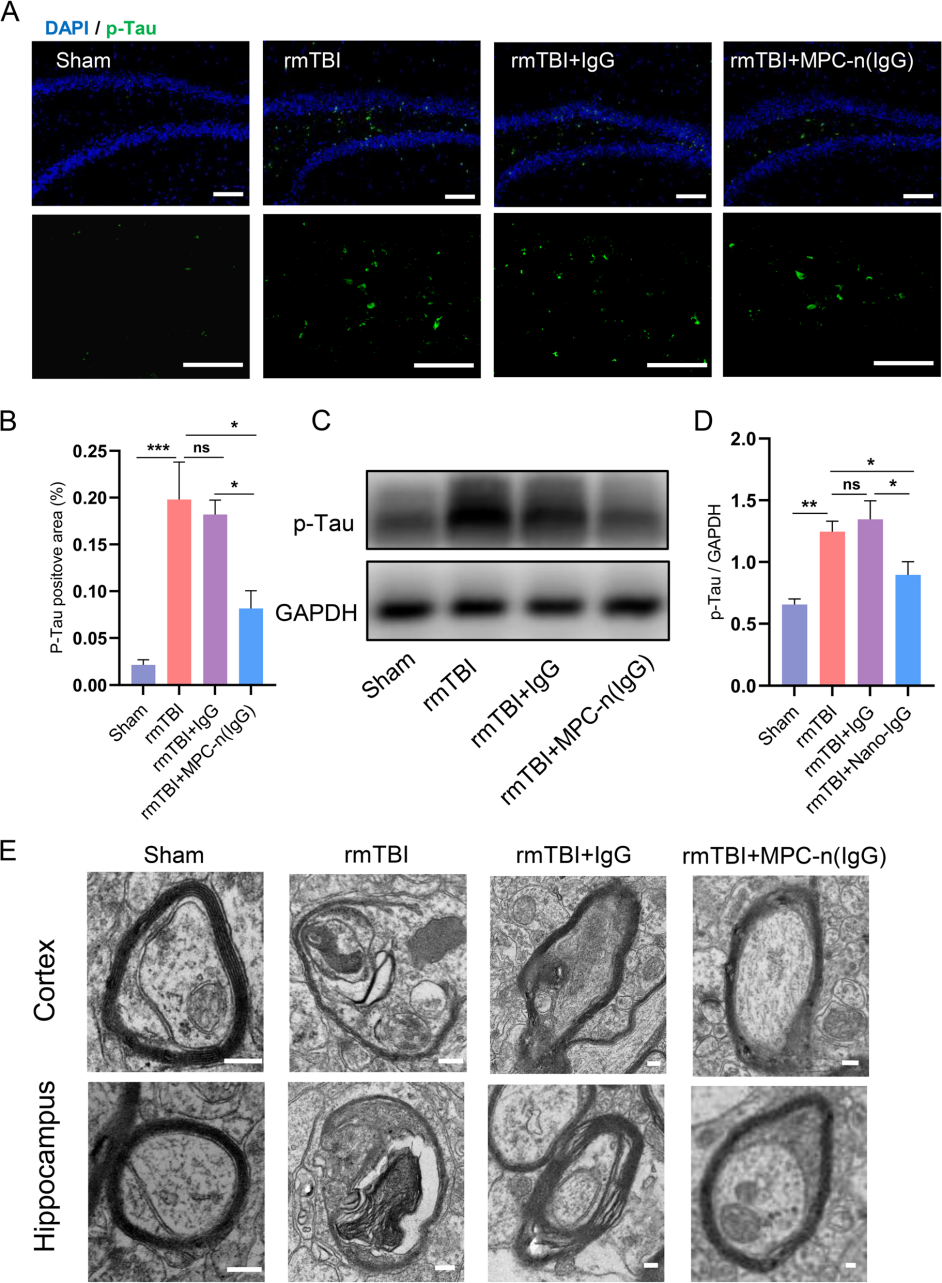
Administration of MPC-n (IgG) alleviates p-Tau deposition and myelin injury. **A**, **B** Immunofluorescence staining of p-Tau in the hippocampus. Scale bar = 50 μm. (*n* = 5–6). **C**, **D** Western blot quantification of p-Tau in the hippocampus. (*n* = 5–6). **E** The ultrastructure of myelin of the cortex and hippocampus was observed at 28 DPI under TEM. Scale bar = 200 nm

Tau protein is an axonal protein that can stabilize and bundle microtubules [33]. However, excessive phosphorylation of Tau protein results in its shedding from the axon and formation of neurofilament tangles, which was considered a key factor in rmTBI-related dementia [34, 35]. The expression of p-Tau protein was observed by immunofluorescence staining, which suggested that p-Tau protein was mainly deposited in the hippocampus (Fig. 4A). Meanwhile, MPC-n (IgG) markedly reduced the deposition of p-Tau in the hippocampus of mice (Fig. 3A, B), which was in line with the result of Western Blot (Fig. 3C, D). In mTBI, shearing and strain forces can directly contribute to multifocal axonal injury [36]. Myelin surrounding the axons of neurons acts as an insulator and increases the conduction speed of nerve impulses, as well as protecting the axons. TEM was used to observe the changes in the ultrastructure of the myelin sheath. As the TEM displayed, the lamellar structure of the myelin sheath was destroyed and the cytoplasm of the neuron was dissolved in both cortex and hippocampus of rmTBI, while MPC-n (IgG) obviously amended the myelin injury compared with free IgG (Fig. 4E).

Taken together, MPC-n (IgG) can more effectively remove p-Tau deposition and remit myelin degeneration than free IgG.

### MPC-n(IgG) suppresses the neuro-inflammatory response

**Fig. 5:**
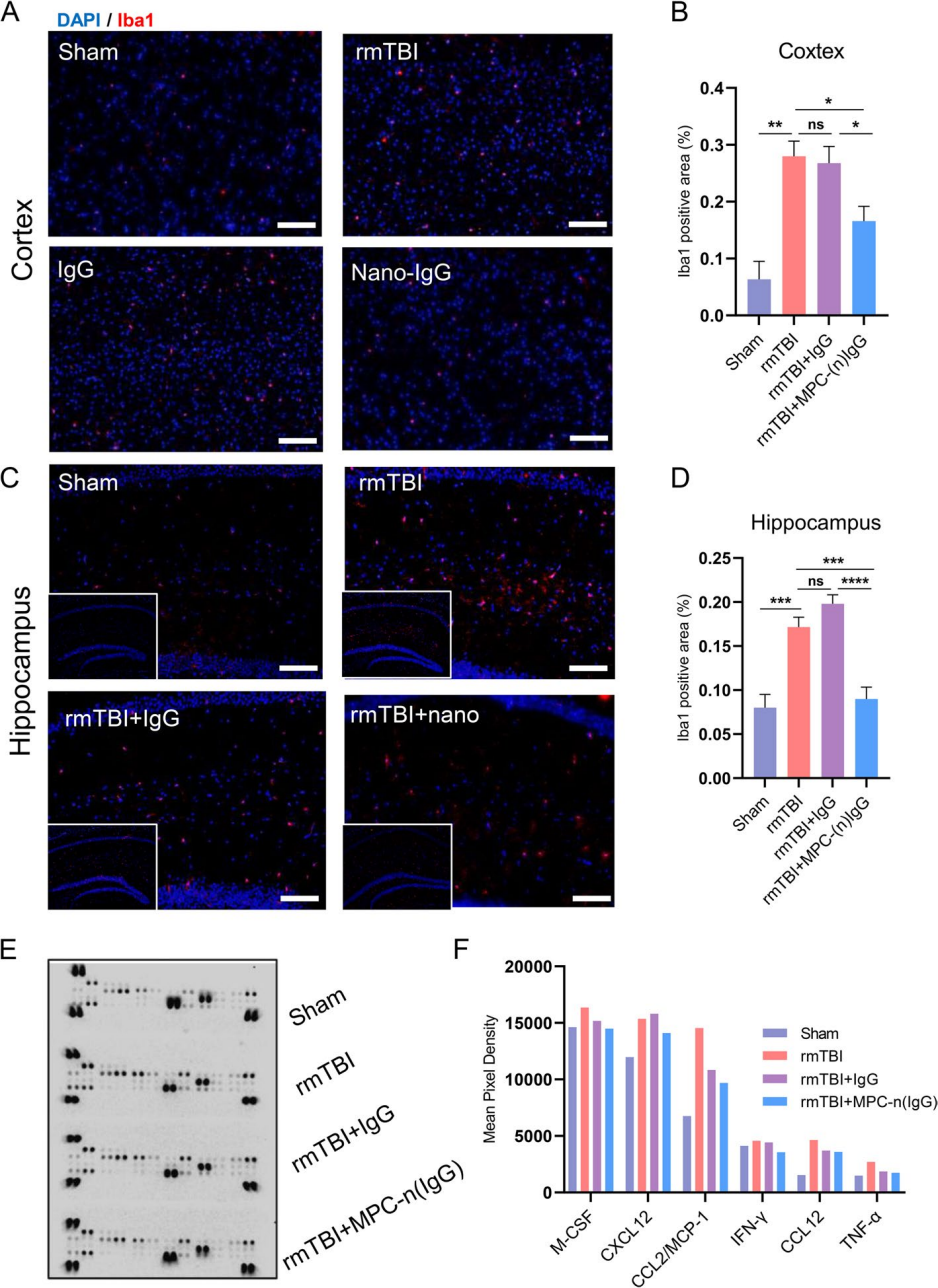
MPC-n (IgG) suppresses the activation of microglia and the release of pro-inflammatory factors. **A**, **B** Immunofluorescence staining of activated microglia in the cortex and **C**, **D** hippocampus. Scale bar = 50 μm (*n* = 5–6). **E**, **F** Array quantification of cytokine quantification indicated the decrease of M-CSF, CXCL12, CCL2/MCP-1, IFN-γ, CCL12, and TNF-α after the treatment of MPC-n (IgG)

Continuous chronic neuro-inflammation often leads to secondary injury and neurodegeneration such as mild cognitive impairment [37, 38]. Microglia acts as the first line of defense in the immune response in the central nervous system, and its activation can last for several months [39]. To evaluate the state of microglia, immunofluorescence was used to detect the expression of activated microglia marker Iba1 in the cortex and hippocampus at 28 DPI. The results exhibited that MPC-n (IgG) was more effective in restraining microglia activation in both the cortex (Fig. 5A, B) and hippocampus (Fig. 5C, D). In addition, cytokines were quantified by Array at 42 DPI, which demonstrated the descent of pro-inflammatory factors under the treatment of MPC-n (IgG), including M-CSF, CXCL12, CCL2/MCP-1, IFN-γ, CCL12, and TNF-α (Fig. 5E, F).

All these results proved that MPC-n (IgG) showed a higher capacity of anti-inflammatory compared with free IgG.

### RNA sequencing reveals the mechanism of MPC-n (IgG) treatment in rmTBI

**Fig. 6:**
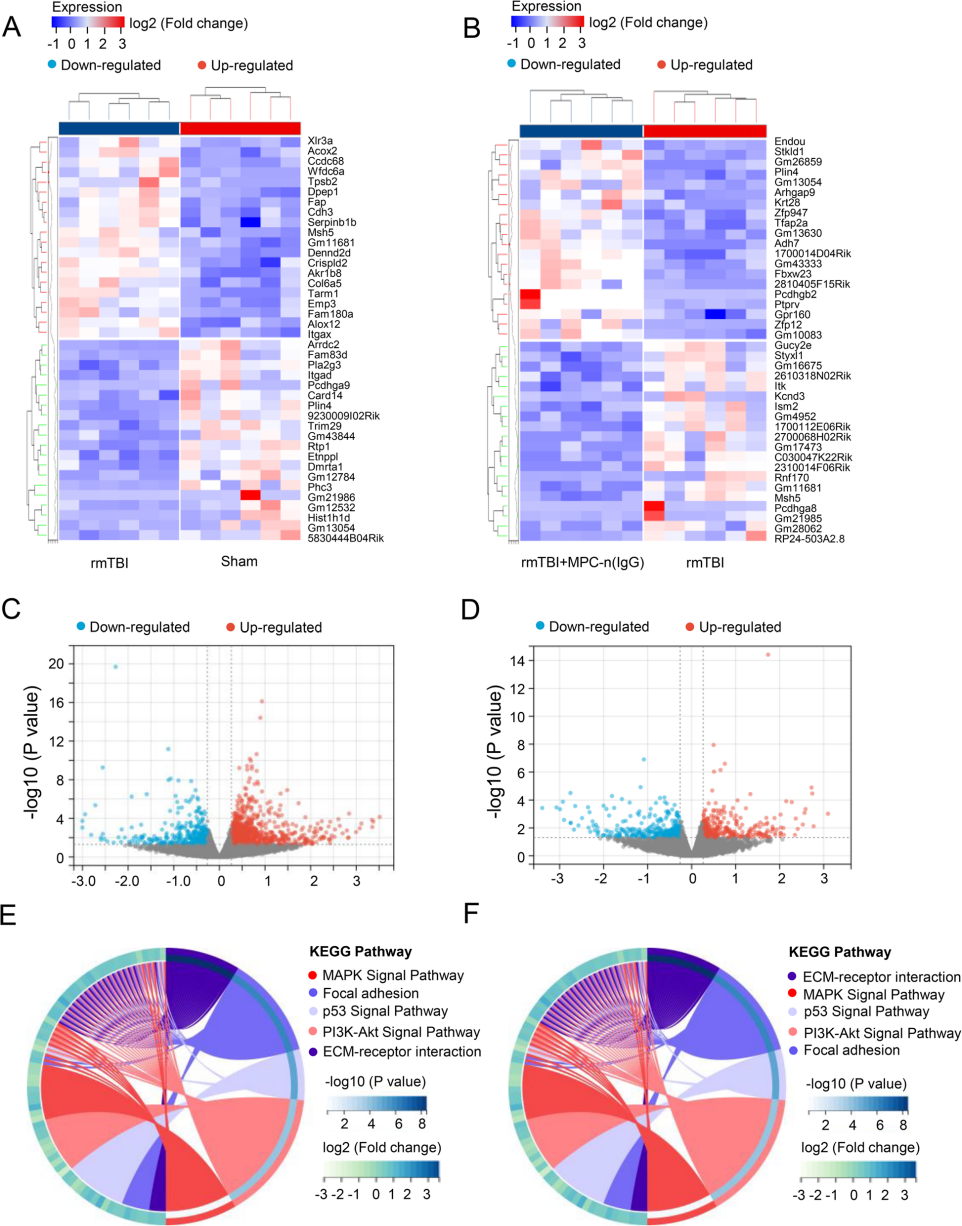
RNA Sequencing reveals the mechanism of MPC-n (IgG) treatment in rmTBI. **A** Heatmap of differential genes, **C** differential gene volcano plot, **E** KEGG circle diagram of differential genes in MAKP pathway between the rmTBI and sham groups. **B** Heatmap of differential genes, **D** differential gene volcano plot, **F** KEGG circle diagram of differential genes between the MPC-n (IgG) and rmTBI groups

In the differential analysis, the results showed the differential genes between the rmTBI group and the sham group (Fig. 6A, C), including 3,574 differential genes, 1794 upregulated genes, and 1780 downregulated genes. Compared with the MPC-n (IgG) group and the rmTBI group, a total of 1625 differential genes were found, including 811 upregulated genes and 814 downregulated genes (Fig. 6B, D). The completed differential gene profiles can be found in Supplementary Tables S1 and S2.

Functional enrichment analysis demonstrated that the changed pathways in the rmTBI group were mainly MAPK signaling pathway, PI3K-Akt pathway, and cell matrix-related pathways compared with the sham group (Fig. 6E). Interestingly, MAPK signaling pathway was also enriched in the MPC-n (IgG) and rmTBI groups (Fig. 6F). Previous studies have shown that MAPK pathway could play an important role in rmTBI [40, 41]; thus, we marked the differential genes in MAPK pathway. As displayed in Additional file 2: Fig. S2A and B, p-p38 MAPK pathway was the most critical gene which upregulated in the rmTBI group compared with the sham group and was lessened in the MPC-n (IgG) group, suggesting that p-p38 MAPK was an underlying treatment target of MPC-n (IgG).

### MPC-n (IgG) downregulates the p-p38 MAPK pathway in rmTBI mice

It was reported that the p38-MAPK pathway contributed to the phosphorylation of Tau protein and synaptic damage [42, 43], and a clinical study showed that the p38α-MAPK inhibitor could improve memory function in AD patients [44]. In addition, the phosphorylation of MAPK in neurons may be a kind of stress response, which turns to promote the activation of microglia and the release of pro-inflammatory factors [45].

**Fig. 7:**
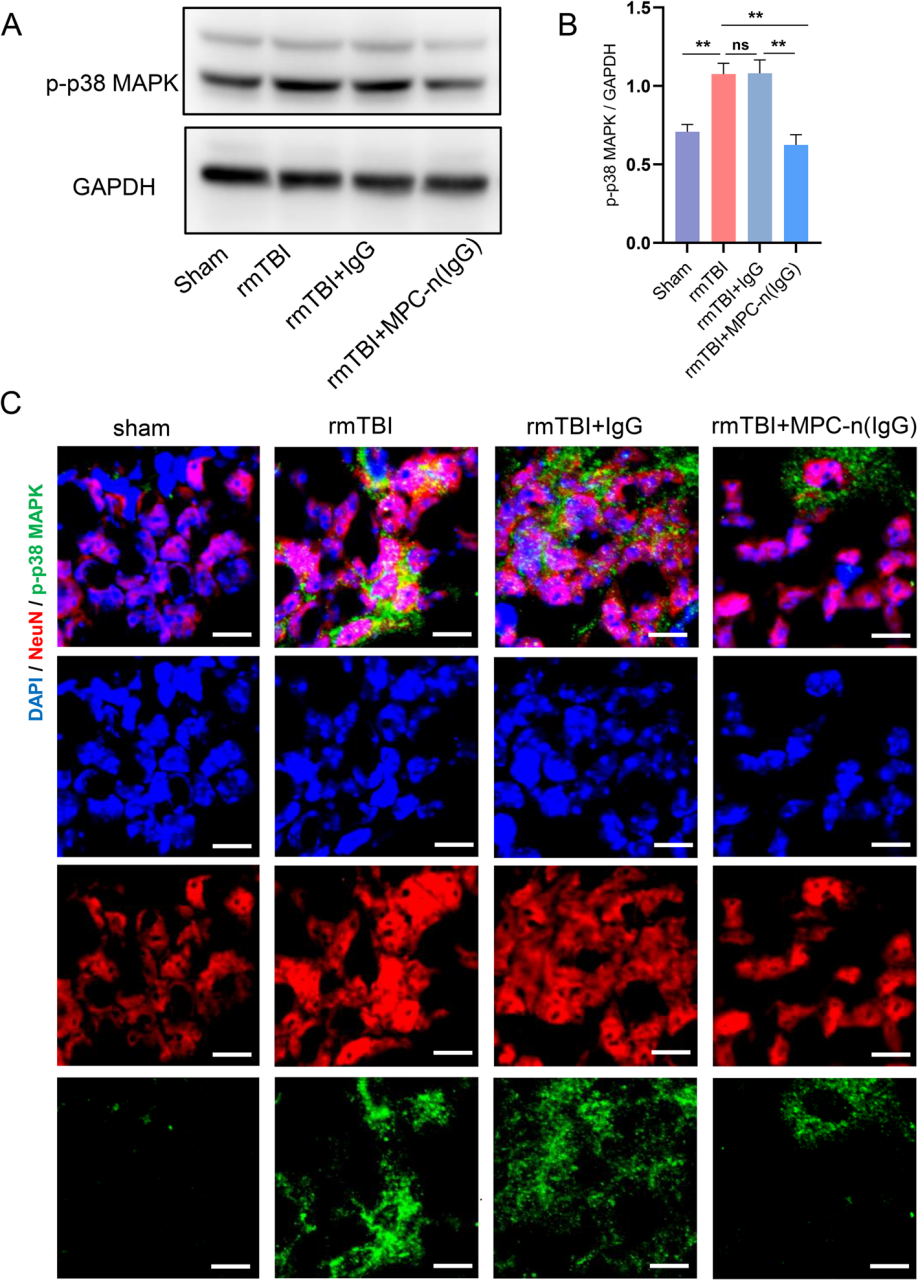
MPC-n (IgG) downregulates p-p38 MAPK pathway in rmTBI mice. **A** Western blot quantification showed that MPC-n (IgG) reduced the expression of p-p38 MAPK in the hippocampus in rmTBI mice (*n* = 5). **B** Immunofluorescence staining illustrated that p-p38 MAPK co-localized with hippocampal neurons. Scale bar = 10 μm

**Fig. 8:**
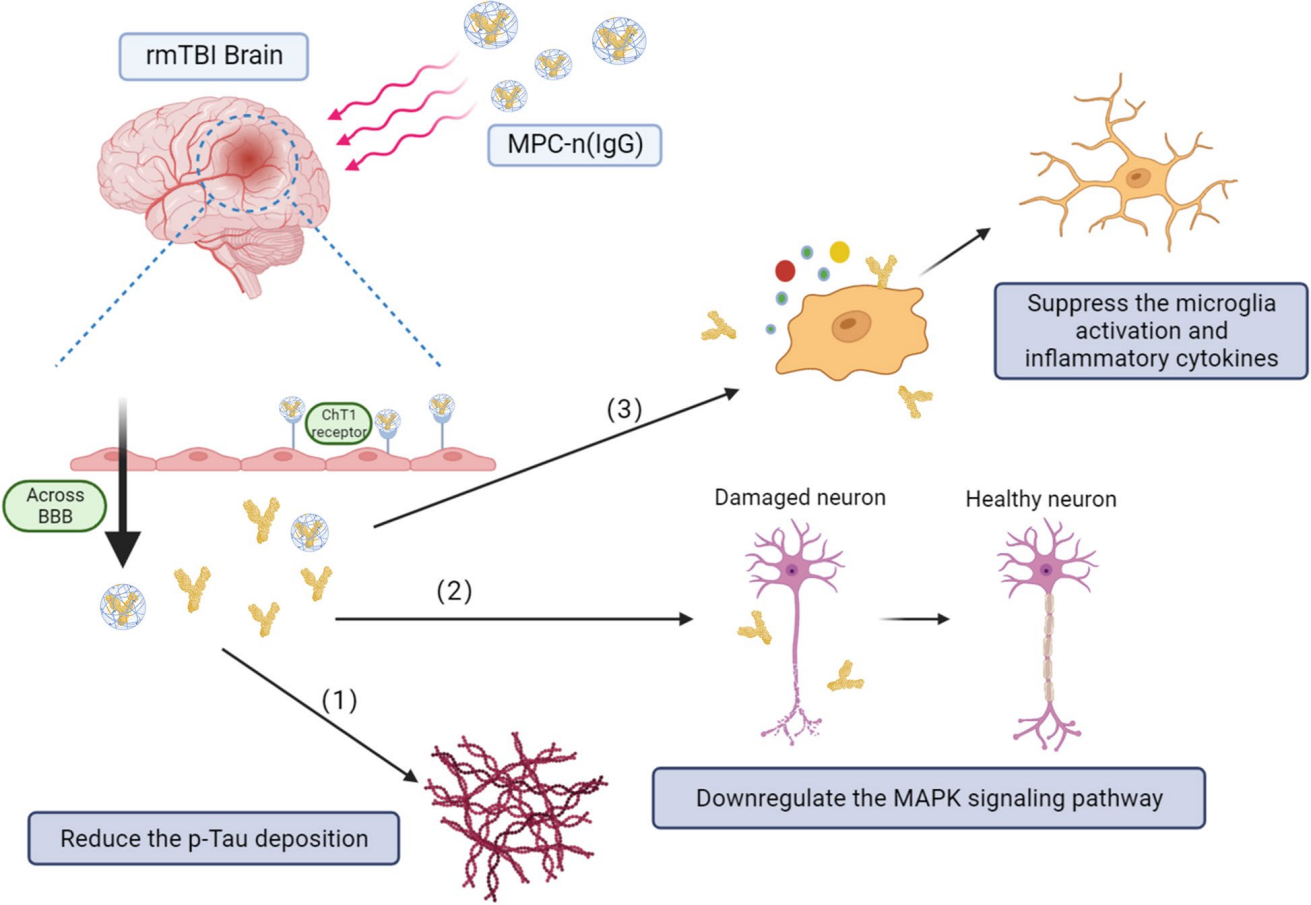
The mechanism diagram of MPC-n (IgG) treatment in rmTBI

According to the sequencing results, we further verified the expression of MAPK pathway in neurons. Western Blot showed that MPC-n (IgG) could effectively reduce the expression of p-p38 MAPK in the hippocampus of rmTBI mice (Fig. 7A, B), and immunofluorescence illustrated that p-p38 MAPK co-localized with neurons in the hippocampus (Fig. 7C), suggesting MPC-n (IgG) can act directly on neurons to exert therapeutic effects (Fig. 8).

## Discussion

Mild traumatic brain injury (mTBI) is the most common subtype of TBI, often presenting with dizziness, headache, and cognitive deficits. Numerous studies have shown that military personnel and contact sports athletes who have undergone rmTBI are at high risk of CTE and advanced dementia [46]. Nevertheless, the lack of effective biological indicators and diagnostic criteria for CTE in clinical practice gaps the difficulty in the treatment.

IgG has been widely used in clinical for more than a century as a superior immunomodulatory agent. High-dosage IgG is approved by the Food and Drug Administration (FDA) as an anti-inflammatory and immune-modulator for several autoimmune diseases such as chronic inflammatory demyelinating polyneuropathy (CIDP) and multifocal motor neuropathy (MMN) [8]. Besides, some experimental studies have shown that IgG exhibits a prominent neuronal protective potential in the treatment of TBI and stroke by removing the C3 complement and downregulating the toll-like receptor of neurons [9, 10, 47]. In recent years, phase II and III clinical trials have been carried out in AD patients, but the effect is not satisfactory. The results suggested that IgG can only remit brain atrophy and cognitive impairment in mild AD patients in a short course, and there was no significant improvement in the long term [12]. In another trial, the AD patients who received IgG did not show a beneficial outcome on cognition but occurred with more systemic responses such as chills and rashes. What is noteworthy is that focused ultrasound (FUS) can temporarily open the BBB and contribute to the specific target of IgG to the hippocampus to reduce amyloid plaque pathology and pro-inflammatory factors [48]. These promising results indicated the significance of facilitating the targeting efficiency of IgG in the treatment of cognitive disorders.

MPC-nanocapsules are composed of PMPC polymer shells and protein cargo. Due to the protection of nanocapsules, the protein can avoid rapid degradation and improve the transportation efficiency of BBB [49, 50]. Our previous studies have clarified that MPC-n (IgG) can be taken up from the peripheral blood by ChT1 receptors of endothelial cells and transported to the brain parenchyma, thereby improving cerebral infarction, neurological deficits and neuro-inflammation after stroke.

As previously mentioned, the association between TBI and cognitive deficits has been expounded. Two main hypotheses have been proposed regarding the mechanism of increased risk, one that TBI decreases cognitive reserve and the second that TBI directly initiates Tau and Aβ pathophysiology processes in dementia [51]. In this study, we administrated low-dosage MPC-n (IgG) (120 mg/kg) and high-dosage free IgG (600 mg/kg) respectively after rmTBI and found that MPC-n (IgG) more significantly improved the cognitive function, p-Tau deposition, and myelin damage of rmTBI mice. It is also notable that the efficacy at42 DPI was more obvious than that at 28 DPI, suggesting that MPC-n (IgG) had a continuous therapeutic effect on the long-term prognosis of rmTBI. In addition, the inflammatory response is an important pathophysiological event in TBI, which may affect outcomes in various ways. Microglia are rapidly activated after brain injury and can persist for months [52, 53]. Our study demonstrated that MPC-n (IgG) also was effective in inhibiting microglial activation and the release of inflammatory factors.

Finally, RNA from the hippocampus at 28 DPI was sequenced to further explore the therapeutic mechanism of MPC-n (IgG). The sequencing results indicated that the mitogen activated protein kinase (MAPK) signal pathway could be an effective target for MPC-n (IgG) treatment. MAPK is an important transmitter of signals from the cell surface to the interior of the nucleus, which can be divided into four subtypes: ERK, P38, JNK, and ERK5. A clinical study of AD showed that the expression MAPK/ metabolism pathway was negatively related to cognitive performance [54]. Another study confirmed that knockdown of p38α-MAPK in AD mice reduced p-Tau load, enhanced synaptic plasticity, and improved cognitive function [55]. IgG has been reported to improve cognition by modulating intracranial and peripheral immunity as well as Aβ pathology [11], but the main mechanism of IgG in the rmTBI was only involved in anti-inflammatory therapy [56]. In our study, we determined that p-p38 MAPK was significantly upregulated after rmTBI and obviously diminished by MPC-n (IgG) using Western blot. Meanwhile, immunofluorescence staining exhibited that p-p38 MAPK could co-localize with neurons, supporting that MPC-n (IgG) may play a neuroprotective role by suppressing the expression of p-p38 MAPK in neurons.

In summary, we proposed an efficient drug delivery system that significantly promoted IgG accumulation in the brain parenchyma of rmTBI mice. Compared with free IgG, MPC-n (IgG) improved cognitive impairment and alleviated chronic inflammation after rmTBI.

## Conclusion

In this study, we applied MPC-capsuled IgG for the treatment of rmTBI-associated cognitive impairment and obtained promising results. We verified that MPC-n (IgG) has a superior ability of BBB penetration and specific target and distinctly improved cognitive function recovery and inhibited neuro-inflammatory response compared with free IgG. Moreover, RNA sequencing revealed that MAPK was a potential target in the clinical therapy of rmTBI-related cognitive deficits. Our study may shed a light on the treatment of cognitive dysfunction and provide evidence for the application of low dosage-IgG.

## Supplementary Information

**Additional file 1: Figure S1. **Characteristics of MPC-n.1H NMR spectra of, IgG, polymer gels without IgG, and MPC-nrecorded in D2O at a concentration of 10 mg/mL.UV-vis spectra of IgG, polymer gels without IgG and MPC-n.The stability of MPC-nin H2O, PBS, DMEM, and serum at 37°C, determined by monitoring particle sizefor 48 h.

**Additional file 2: Figure S2. **Differential genes altered in MAPK pathway in all groupsVisualization of differential genes in MAKP pathway between the rmTBI and sham groups,visualization of differential genes in MAKP pathway between the MPC-nand rmTBI groups.

**Additional file 3: Table S1. **The number of experimental animals.

**Additional file 4: Table S2. **All differentially expressed genes between the rmTBI group and the sham group.

**Additional file 5: Table S3. **All differentially expressed genes between the MPC-ngroup and the rmTBI group.

**Additional file 6. **Original uncropped Western blots of GAPDH 1.

**Additional file 7. **Original uncropped Western blots of GAPDH 2.

**Additional file 8. **Original uncropped Western blots of p-Tau.

**Additional file 9. **Original uncropped Western blots of p-p38 MAPK.

## Data Availability

The data that support the findings of this study are available from the corresponding author upon reasonable request.

## Abbreviations

AD: Alzheimer's disease
Aβ: Amyloidosis
APM: N-(3-Aminopropyl) methacrylamide hydro-chloride
BBB: Blood-brain barrier
CTE: Chronic traumatic encephalopathy
ChTs: Choline transporter
DLS: Dynamic light scattering
EGDMA: Ethylene glycol dimethyl acrylate
FDA: Food and Drug Administration
FITC: Fluorescein isothiocyanate
FT-IR: Fourier transform infrared
FUS: Focused ultrasound
IgG: Immunoglobulins
MAPK: Mitogen activated protein kinase
MMN: Multifocal motor neuropathy
MPC: 2-Methacryloyloxyethyl phosphorylcholine
MPC-n (IgG): MPC-capsuled immunoglobulins
mTBI: Mild traumatic brain injury
PMPC: Poly 2-methacryloyloxy ethyl phosphorylcholine
p-Tau: Phosphorylated-Tau
rmTBI: Repetitive mild traumatic brain injury
TBI: Traumatic brain injury
TEM: Transmission electron microscope
TEMED: Tetramethylethylenediamine
