# Supplementary figures and images for: MPC-n (IgG) improves long-term cognitive impairment in the mouse model of repetitive mild traumatic brain injury
Source: BMC Med. 2023 May 30;21:199. doi: 10.1186/s12916-023-02895-7 (PMC10228048; doi:10.1186/s12916-023-02895-7)

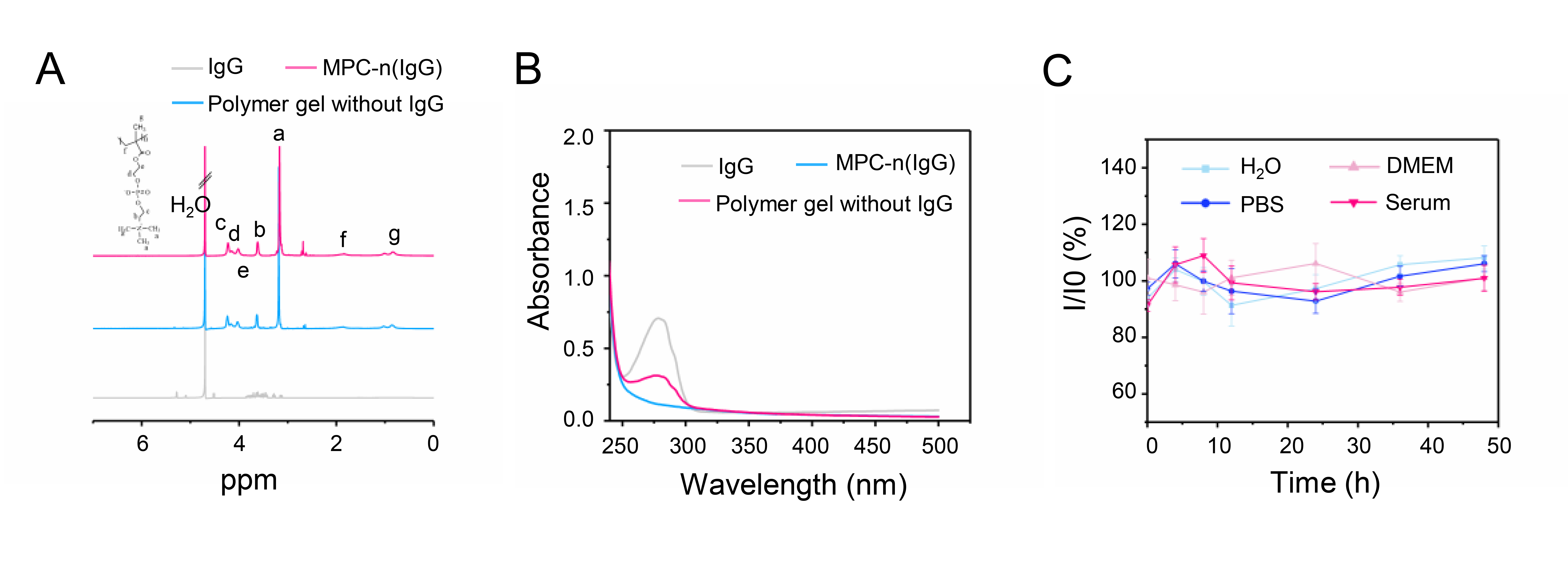

Supplement: Supplementary file 1 — Additional file 1: Figure S1. Characteristics of MPC-n.1H NMR spectra of, IgG, polymer gels without IgG, and MPC-nrecorded in D2O at a concentration of 10 mg/mL.UV-vis spectra of IgG, polymer gels without IgG and MPC-n.The stability of MPC-nin H2O, PBS, DMEM, and serum at 37°C, determined by monitoring particle sizefor 48 h. [file 12916_2023_2895_MOESM1_ESM.tif]

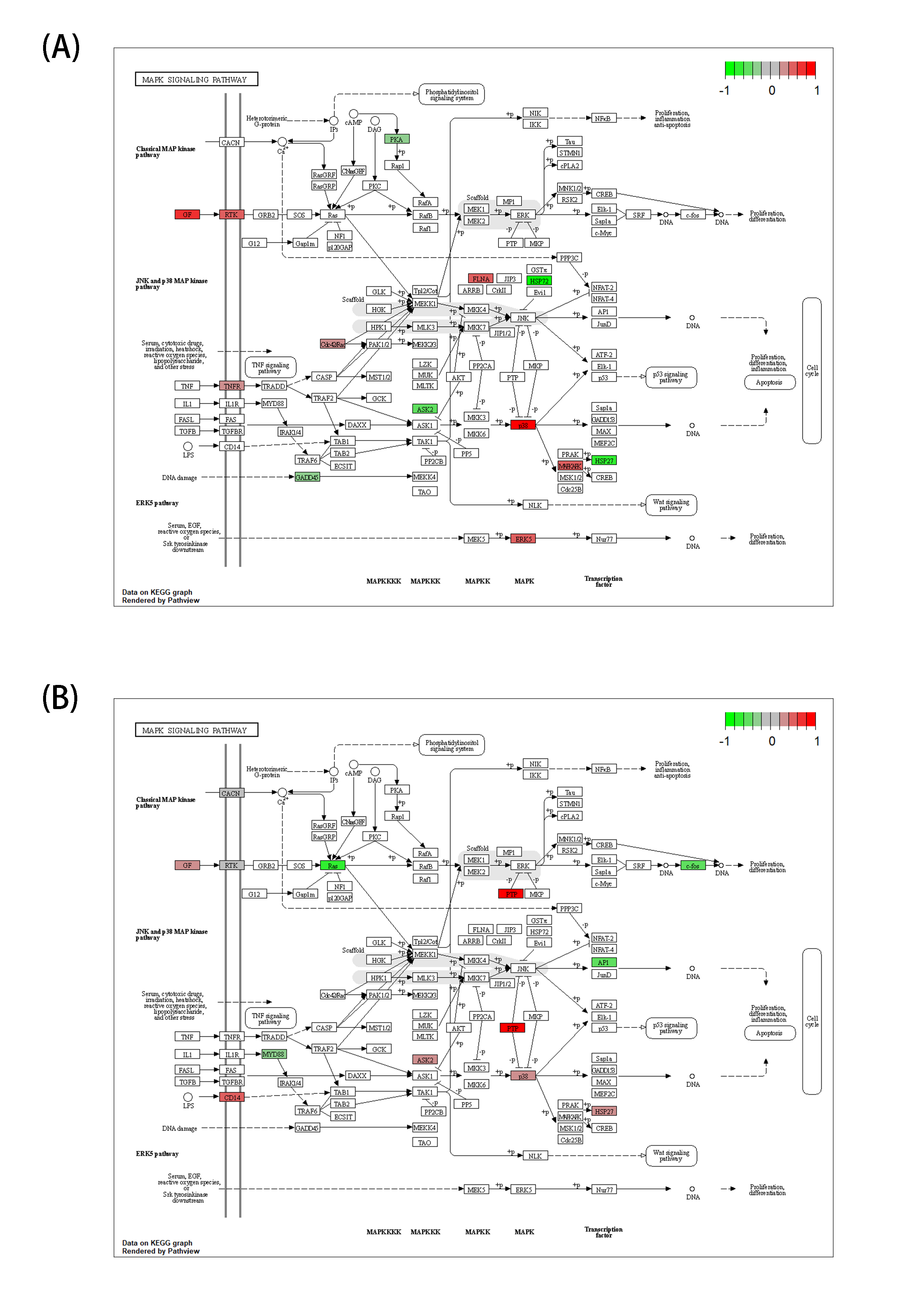

Supplement: Supplementary file 2 — Additional file 2: Figure S2. Differential genes altered in MAPK pathway in all groupsVisualization of differential genes in MAKP pathway between the rmTBI and sham groups,visualization of differential genes in MAKP pathway between the MPC-nand rmTBI groups. [file 12916_2023_2895_MOESM2_ESM.tif]

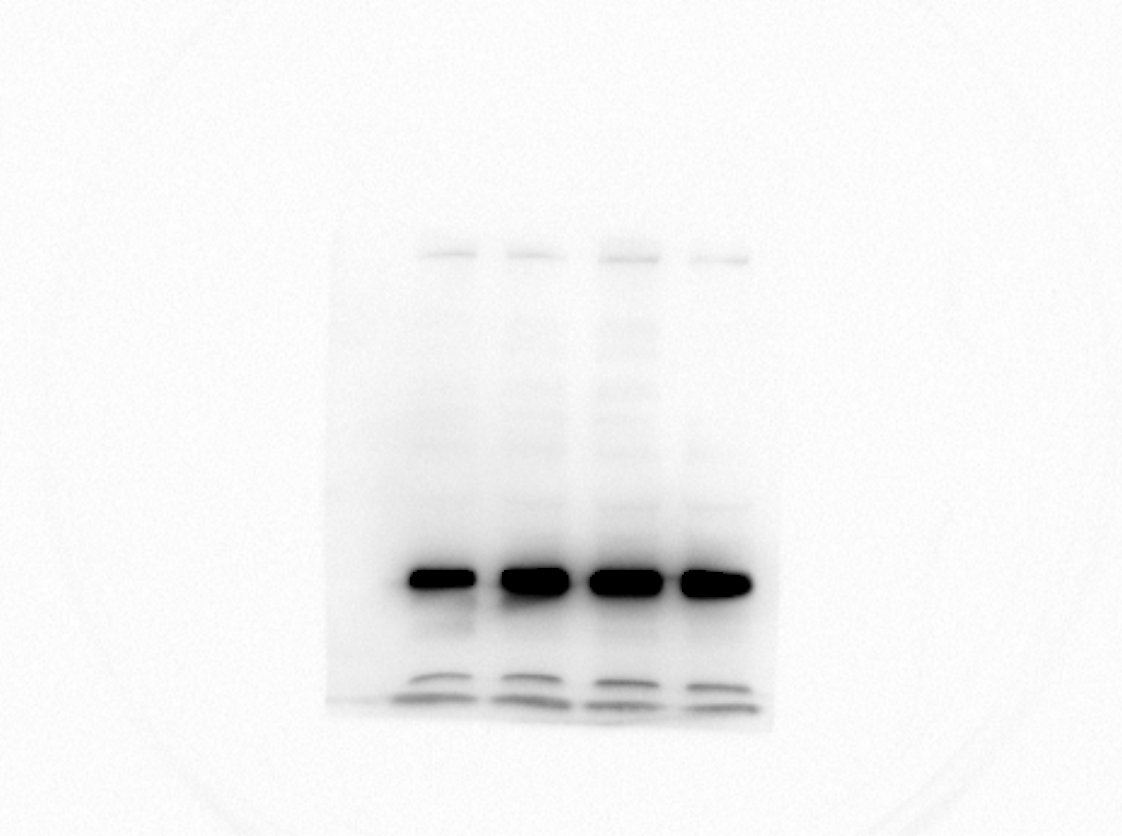

Supplement: Supplementary file 6 — Additional file 6. Original uncropped Western blots of GAPDH 1. [file 12916_2023_2895_MOESM6_ESM.tif]

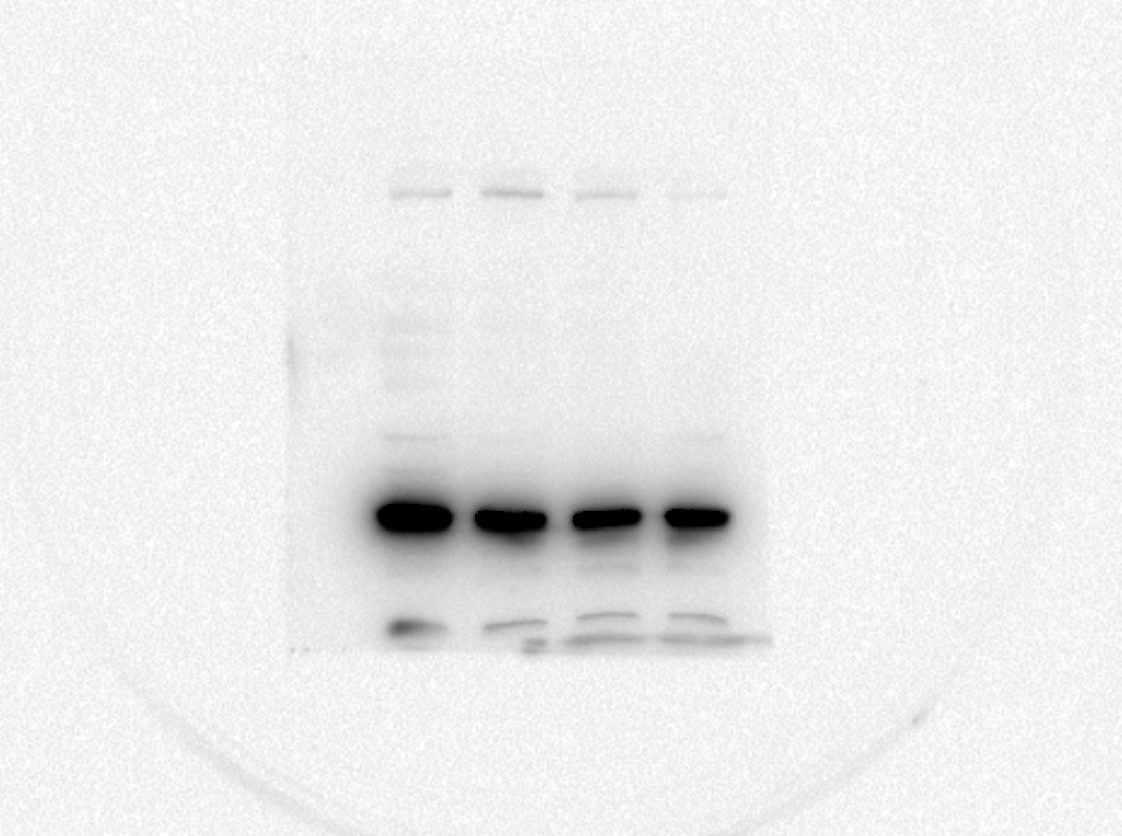

Supplement: Supplementary file 7 — Additional file 7. Original uncropped Western blots of GAPDH 2. [file 12916_2023_2895_MOESM7_ESM.tif]

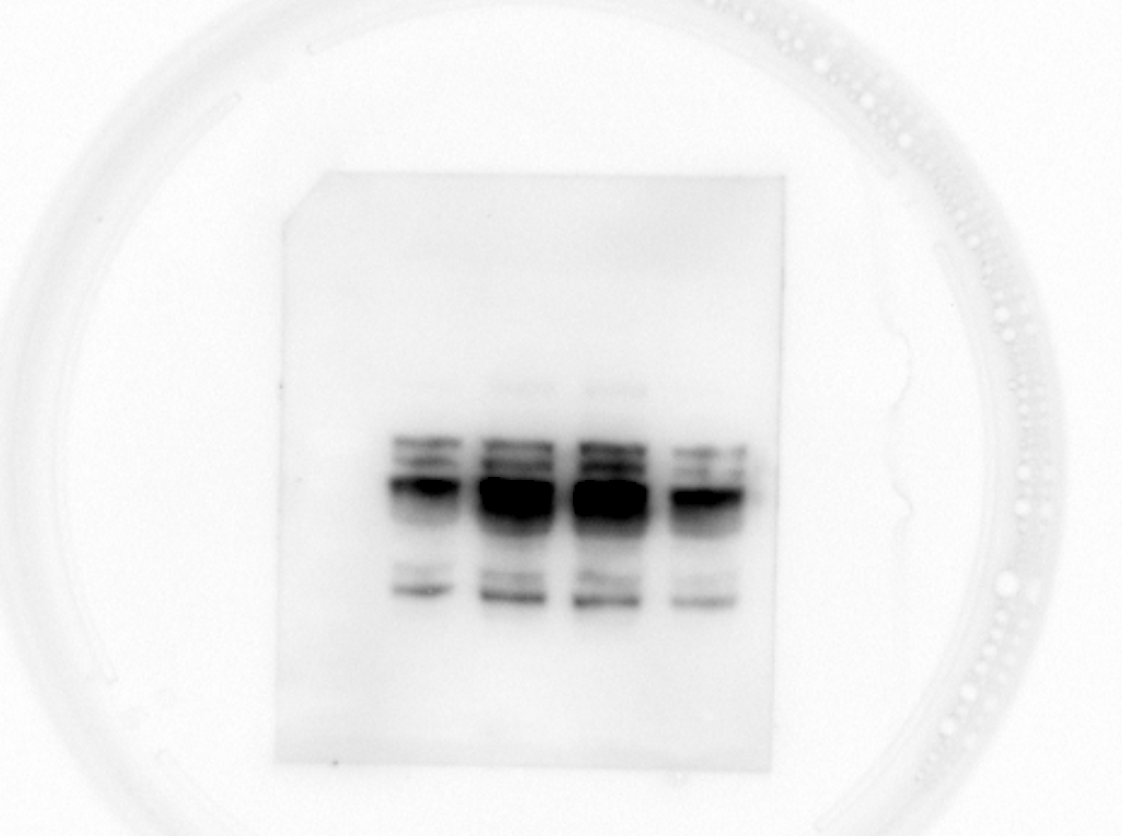

Supplement: Supplementary file 8 — Additional file 8. Original uncropped Western blots of p-Tau. [file 12916_2023_2895_MOESM8_ESM.tif]

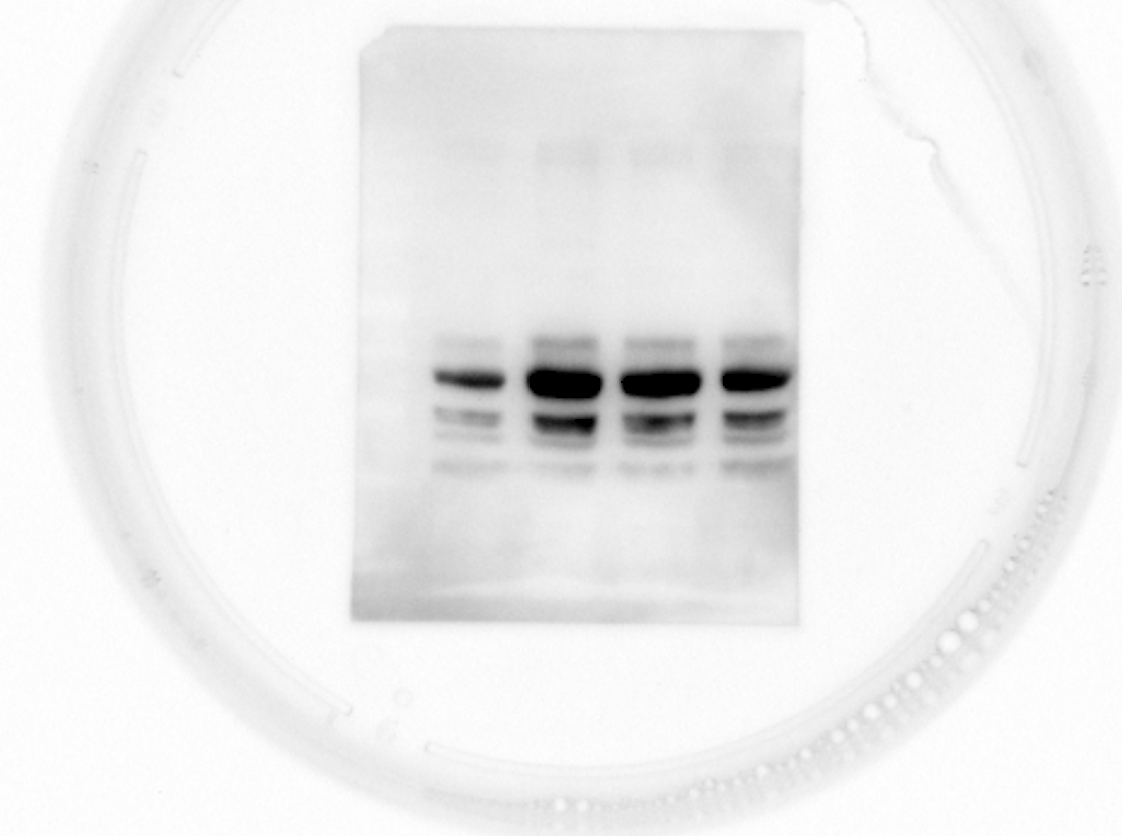

Supplement: Supplementary file 9 — Additional file 9. Original uncropped Western blots of p-p38 MAPK. [file 12916_2023_2895_MOESM9_ESM.tif]
