# Supplementary material for: MPC-n (IgG) improves long-term cognitive impairment in the mouse model of repetitive mild traumatic brain injury
Source: BMC Med. 2023 May 30;21:199. doi: 10.1186/s12916-023-02895-7 (PMC10228048; doi:10.1186/s12916-023-02895-7)
Supplement: Supplementary file 3 — Additional file 3: Table S1. The number of experimental animals. [file 12916_2023_2895_MOESM3_ESM.docx]

| **Experiments** | **Animal number of each group** | **Total number** |
| --- | --- | --- |
| In vivo distribution, imaging, and quantification | 3 mice each group | 12 mice |
| Immunofluorescence staining | 5 sham, 5 rmTBI, 5 rmTBI + IgG, and 6 rmTBI + MPC-n (IgG) | 21 mice |
| Western Blot | 5 sham, 6 rmTBI, 6 rmTBI + IgG, and 6 rmTBI + MPC-n (IgG) | 23 mice |
| Transmission Electron Microscope | 1 mouse each group | 4 mice |
| Magnetic resonance imaging | 5 sham, 6 rmTBI, 6 rmTBI + IgG, and 6 rmTBI + MPC-n (IgG) | 23 mice |
| Morris water maze test | 8 mice each group | 32 mice |
| Cytokine quantification by Array | the MRI mice were used | — |
| RNA sequencing | 6 mice each group | 24 mice |
| **Total number of all experiments** | **139 mice** |  |

**Table S1: The number of experimental animals**
